# Machine learning applied to retinal image processing for glaucoma detection: review and perspective

**DOI:** 10.1186/s12938-020-00767-2

**Published:** 2020-04-15

**Authors:** Daniele M. S. Barros, Julio C. C. Moura, Cefas R. Freire, Alexandre C. Taleb, Ricardo A. M. Valentim, Philippi S. G. Morais

**Affiliations:** 1 Laboratory of Technological Innovation in Health, Federal University of Rio Grande do Norte, Natal, Brazil; 2 Federal University of Goias, Goiania, Brazil

**Keywords:** Machine learning, Deep learning, Retinal image processing, Glaucoma, Classification

## Abstract

**Introduction:**

This is a systematic review on the main algorithms using machine learning (ML) in retinal image processing for glaucoma diagnosis and detection. ML has proven to be a significant tool for the development of computer aided technology. Furthermore, secondary research has been widely conducted over the years for ophthalmologists. Such aspects indicate the importance of ML in the context of retinal image processing.

**Methods:**

The publications that were chosen to compose this review were gathered from Scopus, PubMed, IEEEXplore and Science Direct databases. Then, the papers published between 2014 and 2019 were selected . Researches that used the segmented optic disc method were excluded. Moreover, only the methods which applied the classification process were considered. The systematic analysis was performed in such studies and, thereupon, the results were summarized.

**Discussion:**

Based on architectures used for ML in retinal image processing, some studies applied feature extraction and dimensionality reduction to detect and isolate important parts of the analyzed image. Differently, other works utilized a deep convolutional network. Based on the evaluated researches, the main difference between the architectures is the number of images demanded for processing and the high computational cost required to use deep learning techniques.

**Conclusions:**

All the analyzed publications indicated it was possible to develop an automated system for glaucoma diagnosis. The disease severity and its high occurrence rates justify the researches which have been carried out. Recent computational techniques, such as deep learning, have shown to be promising technologies in fundus imaging. Although such a technique requires an extensive database and high computational costs, the studies show that the data augmentation and transfer learning techniques have been applied as an alternative way to optimize and reduce networks training.

## Objective

This paper describes supervised methods for glaucoma screening in retinal images. The studies reviewed in this article were categorized into deep learning and non-deep learning methods. Hence, its main objective is to evaluate the algorithms recently proposed by different groups, as well as to describe the preeminent steps in the development of an automated diagnosis system. Further, machine learning algorithms can be of significant contribution to the earlier and automated diagnosis of glaucoma, as well as for other abnormal ocular conditions.

## Methods

A literature review aims to synthesize works on a research source to aid further investigations. The methods utilized in the present study were based on the five steps described by Khan et al. [1], as follows: framing questions for a review, identifying relevant work, assessing the quality of studies, summarizing the evidence, and interpreting the findings. Initially, the study objective was defined. Second, the current state-of-the-art of algorithms combining retinal image processing was determined. Then, the sources and criteria were defined in order to include the studies in this review. After that, the most significant works were selected. Furthermore, the analysis of those which presented discussions and perspectives of ML algorithms in the retinal image processing for glaucoma detection and diagnosis occurred. Accordingly, an analysis was performed to determine convergences and divergences amidst studies.

### Data identification and extraction

In this review, the following online databases were considered for the literature research: PubMed, US National Library of Medicine National Institute of Health, IEEE Xplore Digital Library, Science Direct and Scopus.

All the articles were published during the period between January 2014 and August 2019. The search was restricted to the following keywords: “Machine Learning AND Retinal Image”, “Glaucoma AND Machine Learning”, “Optic Disc AND Machine Learning”, “Deep Learning AND Glaucoma”. The keywords search result in 15,228 works. When the exclusion criteria were applied, a total of 110 works remained.

### Selection and exclusion criteria

The selection process was performed according to the following exclusion criteria:

- Studies containing the words “OCT” and “Visual Field” in the title;
- Papers which did not include both “Glaucoma” and “Optic Disc” as keywords;
- Papers which did not include the word “Glaucoma” in the metadata.

In step of study selection, the researches were analyzed through the reading of the abstract, keywords, and methods. As a result, it was possible to acquire the selection criteria of the studies.

The abstracts, keywords and methods of the 110 remaining studies were read in order to analyze their importance and influence. In this stage, the following criteria were applied to the selection of the most significant of those.

- Data acquisition: retinal image;
- Processing techniques: machine learning and deep learning;
- Analyzed eye structure: optic disc (OD);
- Methods that included the image classification process;
- The risk factors in glaucoma detection such as age, family ancestry, and race;
- Journal’s impact factor and paper’s citation number;
- Studies published in proceedings were disconsidered.

According to these criteria, 40 papers were selected for an integral reading. Subsequently, the researches which did not include the classification step (presenting only segmentation) were excluded. In summary, 18 papers were chosen for this review: 10 of those included diverse machine learning approaches and the other 8 comprised methods with deep learning.

## Background

Glaucoma is a neuropathic disease that is marked by ganglion cells degeneration [2, 3]. Thus, an atrophy of the optic nerve fiber is followed by the erosion of the rim tissue, which manifests as a cup enlargement. Currently, the detection of glaucomatous structural damages and changes is one of the most challenging aspects of the disease diagnosis methods [4, 5]. Moreover, glaucoma is generally diagnosed by the analysis of the intra-ocular pressure (IOP)—that should be higher than 22 mmHg without medication—the glaucomatous cupping of the optic disc, and the glaucomatous visual field defects [3].

**Fig. 1:**
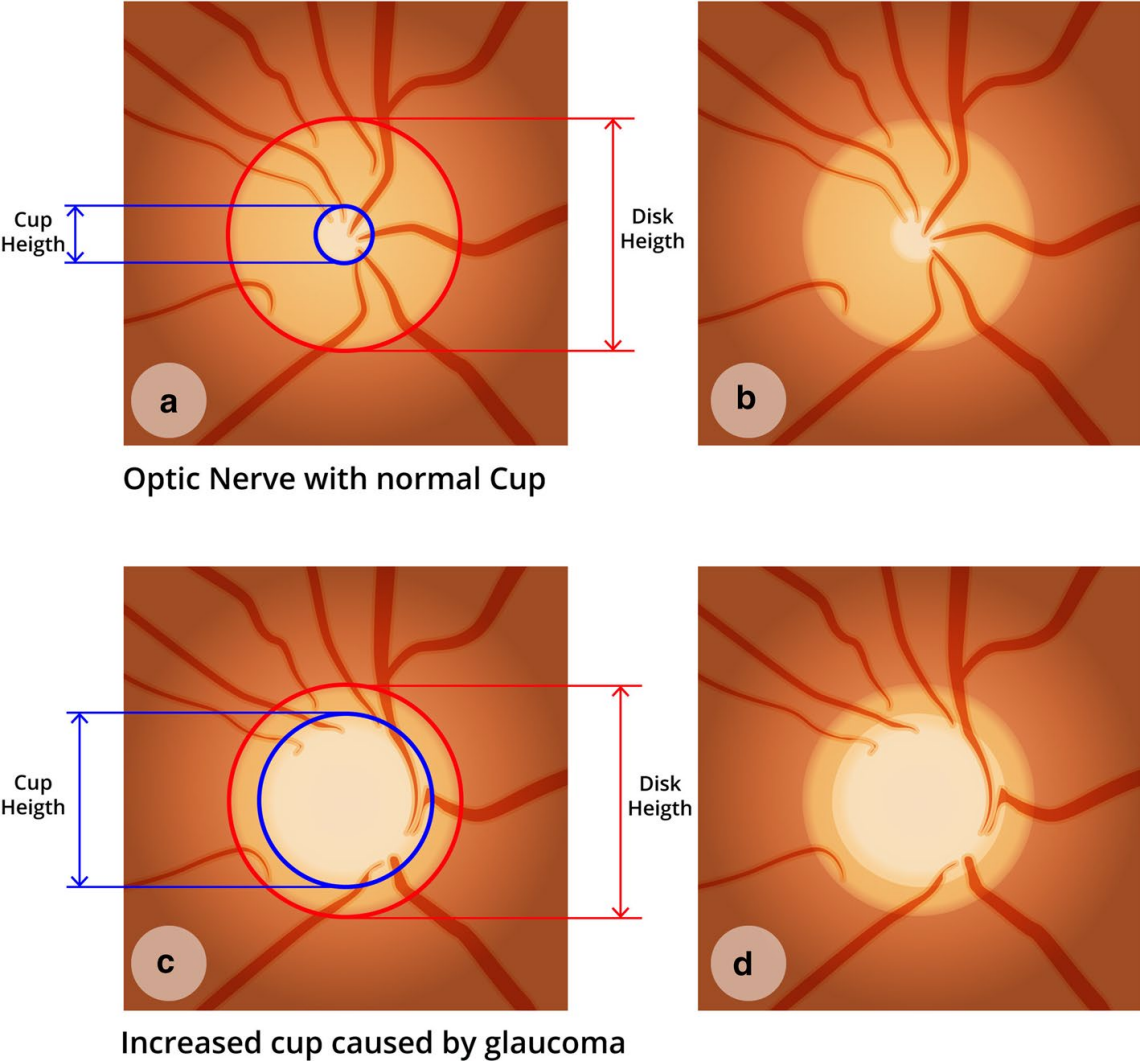
Optic nerve with normal cup and increased cup caused by glaucoma: **a**, **b** optic nerve with normal cup and dimension quotes; **c**, **d** optic nerve with increased cup

One of the greatest challenges regarding glaucoma diagnosis is the asymptomatic aspect of the disease before severe stages. In this way, the number of undiagnosed patients is higher than the number of diagnosed [6]. Yet the size and shape of the optic cup disc is another important aspect to take into consideration during glaucoma diagnosis [7]. Hence, the vertical increase of the cup is a feature of glaucomatous optic neuropathy. By analyzing Fig. 1c, d, it is possible to identify an increase in the cup if compared to Fig. 1a, b. That is a clear glaucoma sign.

The main types of glaucoma can be classified into two categories: primary open-angle glaucoma (POAG) and primary angle-closure glaucoma (PACG) [8]. These two types are considered as the first stages of glaucoma. Still, there is another category called secondary glaucoma, which may be a result of trauma, of some specific types of medications (e.g., corticosteroids), inflammation, tumor, or other abnormal conditions [9].

There is no specific pattern to glaucoma diagnosis. Thus, determining if the patient has the disease becomes a complicated task [10]. Thereupon, longitudinal evaluation and documentation of structural damage to the optic nerve are of paramount importance to the diagnosis [11].

Different technical instruments can be used to aid the diagnosis of glaucoma. For instance, the retinal imaging test, also known as fundus imaging, is widely used among the technology experts [3]. Likewise, there are different methods used for glaucoma screening.

Fundus imaging allows the identification of the main ocular structures, such as the optic disc (OD), optic disc cup (OD-cup), macula region [12], fovea, [13] and blood vessels [14]. This test may also detect abnormal conditions, including microaneurysms, bleeding, exudates, and cotton wool spots [15]. As for the main advantages of retinal imaging, the non-invasiveness, safety, low cost, ease of adoption by ophthalmologists for diagnosis purpose, full coloration, and better detection of disk hemorrhages may be highlighted.

Due to its characteristics, fundus imaging is widely used in the development of diagnostic support systems. Such systems aim to detect and/or diagnose abnormal conditions by analyzing medical images, acting as a second opinion to the health professional [16, 17]. Diagnostic support systems provide clinical decisions to assist physicians regarding their actions to avoid misdiagnosis or incomplete diagnosis.

In the field of ophthalmology, several studies that aim to develop a system for diagnosis support have been carried out. Over the last years, many support systems have been under investigation in the ophthalmic field. Nonetheless, one of the limitations of systems development is the diversity of exams. With reference to retinal images, there are distinct traits due to their variation in the lesion types and to the fact that they differ within themselves [18].

### Statistics

Vision impairment and blindness are significant causes of disability worldwide [19–21]. In prevalence numbers, glaucoma is the second disease that causes such conditions [3]. To examine the global prevalence of POAG and PACG glaucoma variations, as well as to project the number of affected people in 2020 and 2040, Tham et al. [22] developed a systematic review and meta-analysis with data from 50 population-based studies. Thus, according to the results, in 2020 glaucoma will affect around 80 million people worldwide. As for 2040, this number may reach 1115 million [22]. This prediction may reflect the asymptomatic characteristic of the disease.

The incidence rate of many glaucoma types is another important aspect, since it varies in different racial and ethnic groups [23, 24]. Table 1 shows the estimates for each continent. The estimate for Asia and Africa is that more than 85 million people will have glaucoma by 2040.

**Table 1:** Projection of the number of people (aged between 40 and 80 years, in millions) with primary glaucoma in 2020 and 2040

World region^a^	2020^a^	2040^a^
Asia	46.24 (33.08−65.91)	66.83 (48.39–93.77)
Africa	10.31 (6.41−15.28)	19.14 (11.89–28.30)
Europe	7.12 (5.20−9.68)	7.85 (5.76–10.55)
North America	3.94 (2.61−5.72)	4.72 (3.13–6.75)
Latin America and the Caribbean	8.11 (4.46−14.62)	12.86 (7.12–22.85)
Oceania	0.30 (0.16−0.50)	0.42 (0.22–0.69)
Worldwide	76.02 (51.92–111.7)	111.82 (76.50–162.9)

^a^Data from the systematic review by Tham et al. [22]

### Machine learning in the image processing context

The development of new technologies has been demonstrating its relevance for glaucoma diagnosis and treatment. To this extent, machine learning (ML) techniques have proven to be essential for good research results. Still, the main feature of such an approach is the automated task resolution by a smart computational system [25].

ML is a data analysis method that automates the construction of analytical models, which are used in a large range of data types, including images [26]. Mitchell [27] defines machine learning as the ability to improve performance in accomplishing a task through experience.

Systems that use ML can learn from data, identify patterns and make decisions with minimal human intervention. Thus, in the context of diagnosis modeling using the classification paradigm, such a learning process is based on observing data as examples. In this situation, the model is constructed by learning from data along with its annotated labels.

In order to use some ML models when problems in image processing occur, it is necessary to reduce the number of data entries. An image can be transformed into millions of pixels for tasks such as classifications. In this sense, data entry would make processing very difficult. Then, to make it easier, the image is transformed into a reduced set of features. This operation selects and measures the representative properties of raw input data in a reduced form [28]. Moreover, such a set represents the relevant piece of information required to perform a desired task. It can be represented by color, texture, shape or a simple portion of the image [29].

The main purposes of the studies in this field are to identify features to reduce memory and processing time requirements, to eliminate irreversible attributes, and to simplify the generated model. Earlier studies usually applied computer vision methods to manually extract designed features. Accordingly, some methods used non-segmentation-based method, which designs various features, such as entropy [30], wavelet [31] or fractal dimensions [32]. Nonetheless, alternative methods applies a segmentation-based approach, which generates common measures for glaucoma diagnosis.

Image segmentation is the separation of the target region, those corresponding to the object of the real world, from the image background. For this process, Cheng et al. [33] proposed an optic disc and optic cup segmentation system that uses superpixel classification. Additionally, Chrástek et al. [34] developed a method for optic nerve head (ONH) segmentation whose validation was based on morphological operations. In both techniques, the target region is based on the needs of specific applications. Usually, it corresponds to the subjective cognition and the experience of the operator [35].

Over the last years, some reviews were performed in order to describe which automated methods have been applied in glaucoma diagnosis. Diaz-Pinto et al. [36] employed five different deep learning models to assess glaucoma. Yet Almazroa et al. [37] reviewed segmentation methodologies and techniques for the disc and cup boundaries, the structures utilized to help to diagnose glaucoma. While the first study solely focused on deep learning methods, the second employed only heuristic and image processing techniques. On the other hand, in the present review, we have analyzed the supervised methods, categorizing both deep learning and non-deep learning algorithms for glaucoma diagnosis.

**Table 2:** Main studies using features extraction

Methods	Year	Data	Preprocessing	Features Extract	No. of features	Best classifier^a^	Results^a^ (%)		
							Acc	Sp	Sn
Noronha et al. [38]	2014	272	Image resize with interpolation method	Higher order cumulant features	35	NB	92.65	100.00	92.00
Acharya et al. [39]	2015	510	Image resizing with histogram equalization	Gabor transform	32	SVM	90.98	91.63	91.32
Issac et al. [40]	2015	67	Image resizing with statistical features	Cropped input image after segmentation	3	SVM	94.11	90	100
Raja et al. [45]	2015	158	Grayscale conversion and histogram equalization	Hyper-analytic wavelet transformation	16	SVM	90.14	85.66	94.30
Singh et al. [47]	2016	63	N/A	Wavelet feature extraction	18	k-NN	94.75	100	90.91
Maheshwari et al. [30]	2017	488	Grayscale conversion	Variational mode decomposition	4	LS-SVM	94.79	95.88	93.62
Soltani et al. [48]	2018	104	Histogram equalization and noise filtering	Randomized Hough transform	4	Fuzzy logic	90.15	94.80	97.80
Koh et al. [49]	2018	2220	NA	Pyramid histogram of visual words and Fisher vector	4 x 4 (grid)	RF	96.05	95.32	96.29
Mohamed et al. [50]	2019	166	Color channel selection and illumination correction	Superpixel feature extraction module	256	SVM	98.63	97.60	92.30
Rehman et al. [51]	2019	110	Bilateral filtering	Intensity-based statistical features and texton-map histogram	2	SVM	99.30	99.40	96.90

^a^Only the best results obtained in each method were left

k-NN classifier, least-squares support vector machine LS-SVM, random forest RF, naive Bayes NB, support vector machine SVM

## Results

In this section, we present an overview of the existing literature on machine learning algorithms for retinal image processing. Initially, the studies that utilize feature extraction methods are showed (listed in Table 2). Then, deep learning techniques are specifically presented, such as the convolutional neural network (CNN) architectures (as described by Table 3). The methods are approached in the context of glaucoma screening. Finally, all the metrics used to evaluate the results, as well as their values, are summarized in Tables 2 and 3.

**Table 3:** Main studies using deep convolutional network

Methods	Year	Architecture	Metrics (%)		
			Acc	Sp	Sn
Li et al. [55]	2018	Inception-v3	92	95.6	92.34
Fu et al. [58]	2018	Disc-aware ensemble network (DENet)	91.83	83.80	83.80
Raghavendra et al. [62]	2018	Eighteen-layer CNN	98.13	98.3	98
dos Santos Ferreira et al. [63]	2018	U-net for segmentation and fully connected with dropout for classification	100	100	100
Christopher et al. [65]	2018	ResNet50	97	93	92
Chai et al. [68]	2018	MB-NN	91.51	92.33	90.90
Bajwa et al. [69]	2019	Four convolutional layers and fully connected layers	87.40	85	71.17
Liu et al. [72]	2019	ResNet	99.6	97.7	96.2

^a^Only the best results obtained in each method were entered

### Methods using features extraction for glaucoma diagnosis

In 2014, Noronha et al. [38] developed a system using higher order spectra (HOS) cumulants. In the first step, the image was decomposed into projections with the application of Radon transform. These were used to compute high-order statistic moments. Then, its combination constituted the high-order cumulant features. Thereafter, a dimensionality reduction was performed through principal component analysis (PCA), independent component analysis (ICA), and linear discriminant analysis (LDA). As a result, the LDA yielded the highest classification accuracy and its results were applied in a feature ranking method using Fisher’s discrimination index (F). In addition, support vector machine (SVM) and naive Bayes (NB) methods were applied in the classification process. The system was tested in a private database consisting of 272 fundus images. Hence, with the use of a tenfold cross-validation method, 100 images exhibited normal conditions, while 72 and 100 revealed mild glaucoma and moderate/severe glaucoma, respectively.

In 2015, Acharya et al. [39] developed a method using features that were extracted from Gabor Transform. From this procedure, the extracted coefficients were: mean, variance, skewness, kurtosis, energy, and entropies such as Shannon, Rényi, and Kapoor. Then the obtained features were subjected to PCA. Furthermore, feature ranking is another fundamental process to the proposed algorithm, since it allows the most representative features to be selected. Thus, the following were chosen: t-test, Bhattacharyya space algorithm, Wilcoxon test, receiver operating curve (ROC), and entropy ranking methods. The proposed method was tested in a private database consisting of 510 fundus images, with the succeeding classifications: normal (266); mild (72); moderate (86) and severe (86). To split the groups with and without glaucoma, the authors also proposed a numerical risk index for the condition. The SVM and NB were used in the classification process.

Still, in 2015, Issac et al. [40] developed an adaptive threshold-based method to segment the OD and OD-cup for glaucoma diagnosis. First, the ONH region was analyzed. Then, the histogram of the green channel was used for the OD segmentation. The selected features for OD and OD-cup identification were: cup-to-disc ratio (CDR), neuroretinal rim (NRR) area, blood vessel area and the ISNT rule. More details about CDR, NRR, and ISNT rule can be found in the works of Hu et al., Mabuchi et al., Jonas et al. and Poon et al. [41–44]). Moreover, the SVM, the function kernel RBF, and the Artificial Neural Network (ANN) were used as classifications. The private dataset used to test the method was composed by 67 images, 35 being healthy and 32 glaucomatous.

In the same year, Raja et al. [45] proposed a method based on the particle swarm optimization (PSO) and group search optimization (GSO). With the application of the PSO framework, the g-best values were extracted through the population. At this point, the global optimal and potential members were scanned in order to identify better members. The preprocessing was performed with the application of the grayscale conversion and histogram equalization. Regarding the feature extraction step, the hyper-analytic wavelet transform (HWT) and wavelet transform were applied. Such a process extracted the mean, energy, and entropy features that were utilized in the classification process. Later, the method was tested in the RIM-ONE public database [46], and the best results were attained with the SVM classifier.

Following that, in 2016, Singh et al. [47] proposed a method using wavelet feature extraction. Its initial step is characterized by OD segmentation and blood vessel removal. In this method, the largest lighter region is considered the optic disc center. In addition, the wavelet feature extraction process was used in the segmented OD. Thus, the dimensionality reduction utilized PCA, and the normalization process, z-score. For the test and the training process, 63 images from patients aged between 18 and 75 years were utilized. Such a material was acquired from a private database. Still, the classification was performed using the random forest, NB, kNN and Artificial Neural Network (ANN) classifiers. Conclusively, the best results were reached with kNN and SVM.

In 2017, a method that applied a non-stationary classification technique, based on the VMD algorithm to get ten band-limited sub-signals, was developed by Maheshwari et al. [30]. Hence, textural features were extracted from those components in order to measure smoothness, coarseness, and pixel regularity in the images. After that, the normalization step used the z-score. The RelieF algorithm was used to select the discriminatory features, which were fed to least-squares support vector machine (LS-SVM) during the classification process. The training step was performed in a private database consisting of 488 images, in which one half was normal, and the other with glaucoma. In addition, the test was carried out in the RIM-ONE [46] dataset.

One year later, Soltani et al. [48] developed an algorithm for classification that was based on a fuzzy logic approach. Specifically, the algorithm examines risk factors such as age, family heredity, race, and image data. It consists of the following steps: image preprocessing, characterized by noise removal; OD contour detection and identification; and excavation and extraction of key parameters. In this manner, the features were extracted through the randomized Hough transform (RHT). Afterwards, the images were classified, with the implementation of SVM, as normal, glaucoma-suspicious or glaucomatous. The method was performed in a private database consisting of 104 images, in which 46 were glaucomatous and 58, normal.

Also in 2018, Koh et al. [49] developed an algorithm based on the pyramid histogram of visual words (PHOW) and the Fisher vectors. Thusly, this algorithm extracts the PHOW from the background images. Following that, the Gaussian mixture model (GMM) was performed on the training set to obtain a vocabulary to encode the Fisher vectors for training and testing. Then, the features were built with the application of the vectors, which were used as an input for the random forest classifier. Such a method employs a blindfold and tenfold cross-validation techniques for the validation process. Lastly, 2220 images were also acquired from a private database: 553 manifested glaucoma, 346 diabetic retinopathy, 531 age-related macular degeneration, and 790 represented normal conditions.

In the year of 2019, Mohamed et al. [50] developed a cup and disc segmentation method. The image pixels were clustered through the simple linear iterative clustering superpixel technique. During the image preprocessing step, the peak signal-to-noise ratio (PSNR) and contrast-to-noise ratio (CNR) were utilized for quantitative evaluation. Therefore, a Simple Linear Segmentation (SLIC) algorithm was adopted to generate the desired number of superpixels, as well as the adjustable clustering compactness. Following the segmentation process, the mean, variance, kurtosis, and skewness features were extracted with an algorithm denominated as statistical pixel-level (SPL). The SVM was used in the classification step with both linear function kernel and RBF. As for the method testing, it occurred by means of the RIM-ONE database [46].

Lastly, in 2019, Rehman et al. [51] applied a superpixel technique. The image preprocessing constituted three steps: noise removal and OD-edge enhancement, image enhancement and cropping, and cropping around the OD region. The SLIC superpixel method was adopted during the segmentation process. Moreover, the intensity-based statistical features, along with texton-map histogram, characterized the extraction technique. The statistical features extracted from the superpixels were: average, maximum, minimum, median, and mode intensity. In addition, the fractal feature was another feature extractor used. The authors obtained five features with statistical methods, six, with texton histogram, and six more with the fractal method. Then, SVM and RF methods were employed in the classification step. Regarding the test, the chosen methods were the DRIONS [52], the MESSIDOR [53], and the ONHSD [54] datasets. Thereupon, the best result was reached with the RB classifier in the ONHSD database.

### Methods using deep convolutional network architectures for glaucoma diagnosis

In 2018, Li et al. [55] developed a method to evaluate the performance of a deep learning algorithm for the glaucoma optic neuropathy (GON) screening. To this extent, the Inception-v3 [56] was applied in the network architecture with a mini-batch gradient descent of size 32, which was used during the training process. Following that, Adam Optimizer [57] was utilized for the convergence step. The best result was reached with a learning rate equal to 0.002. Further, the method was performed in a private database consisting of 70,000 images. However, only 48,116 of those, that showed a visible optic disc, were used. With the utilization of the ground truth labeled by the medical experts, two detection levels of referable GON were adopted: non-referable GON and referable GON (constituted of suspected and certain GON).

That same year, Fu et al. [58] proposed a Disc-aware Ensemble Network (DENet) for automated glaucoma screening which took into account the global and local image levels. In this manner, the global level was composed of two streams. The first of those streams was defined as a standard classification network using Residual Network (ResNet). As for the second, it was a segmentation-guided network adapted by the U-shape convolutional network [59]. In order to transfer the OD region into polar coordinate system, the local image-level was composed of the standard classification network based on the ResNet and disc polar transformation stream. Such an architecture contains four deep streams: the global image stream, the segmentation-guided network, the disc region stream, and the disc polar stream transfers. All the deep streams outputs were combined for achieving the final screening result. The experiments applied ORIGA [60], Singapore Chinese Eye Study (SCES) [61], and the SINDI private database.

Still, in 2018, Raghavendra et al. [62] designed a method with an 18-layer CNN architecture composed of convolutional and max-pool layers. The classification layer was performed by utilizing the logarithmic soft-max activation function. Then, one output neuron was used to get the probability for each class. Training and testing steps were performed in images from the Kasturba Medical College private database: 589 images were defined as normal and 837, as abnormal. About 70% of the data was randomly adopted for training, and 30% for testing.

In addition, dos Santos Ferreira et al. [63] schemed a method for both image segmentation and classification in 2018. First, a U-net convolutional network was trained to perform the OD segmentation. At this stage, 80% of the data was employed for training and 20%, for testing. Then, the blood vessels were removed using Otsu algorithm. In the following step, an extraction of texture-based features was performed. To describe the texture of the ROI, parameters to calculate the phylogenetic density present in this structure were applied. Furthermore, a neural network implementation, based on the last CNN classification layer, was implemented. It consisted of 100 fully connected layers in which the dropout was equal to 0.5, the net was tested throughout 1,000 epochs, and the learning rate was 1 × 10^−5^. The images used in the test and training steps were acquired from RIM-ONE [46], DRIONS-DB [52] and DRISHTI-GS [64] databases.

In that corresponding year, Christopher et al. [65] evaluated the performance of the deep learning architecture for the GON screening by employing the native and the transfer learning-based methods. Therefore, the images were preprocessed to extract the ONH-centered region. A data augmentation process was applied in which two random orientations and five random crops were employed in each image. Consequently, the dataset was increased by 10 times. What is more, the VGG16 [66], Inception-v3 and ResNet50 [67] architectures were used. Subsequently, the data were partitioned into independent training, validation, and testing sets using an 85–5–10% splitting. During the training process, the tenfold cross-validation technique was utilized. The method was performed in a private database consisting of 14.822 images: 33% were of African descent people. In conclusion, the most satisfactory results were achieved through ResNet50 with transfer learning.

Once more in 2018, Chai et al. [68] proposed a multi-neural network branch (MB-NN) model using a domain knowledge, as well as hidden learning resources. In this way, the following features were extracted: CDR, retinal nerve layer defects (RNFLD), peripapillary atrophy (PPA), OD size, and ISNT rule. The Faster-RCNN deep learning model was adopted in the image segmentation process to obtain these features. Moreover, the applied domain knowledge features were: age, IOP, eyesight, and binary features, such as failing eyesight and eye injuries. CNN’s model was divided into branches, in which the first and the second analyzed the entire image and the disc region. As for the third, it comprehended a fully connected forward neural network in order to deal with domain knowledge features. Further, the training process used the multi-branch neural network (MB-NN) model. The method was performed in a private dataset consisting of 2554 images, in which 1023 indicated glaucoma or other conditions, and 1531 were non-glaucomatous.

A year later, Bajwa et al. [69] developed a two-stage framework for ROI localization and glaucoma classification. Thus, the first stage was performed in two steps. Initially, a semi-automated ground truth (GT) was developed, characterized by a RCNN-based architecture that automatically detected the OD. Next, the classification process was run. The OD was extracted, and the deep network was utilized. Thereupon, the architecture was divided into four convolutional layers: a Max pooling overlapping strides and three fully connected layers. The training, testing, and validation data were acquired from ORIGA [60], HRF [70], and OCT & CFI [71] databases. The most efficient results were achieved by means of cross-validation.

In 2019 as well, Liu et al. [72] established a large-scale database of fundus images for glaucoma diagnosis, also known as the FIGD, and developed convoluted neural networks (GD-CNN) for automatically detecting GON. The network architecture was based on the ResNet [73]. The database was formed by 274.413 fundus images which were obtained from the Chinese Glaucoma Study Alliance.

## Discussion

**Fig. 2:**
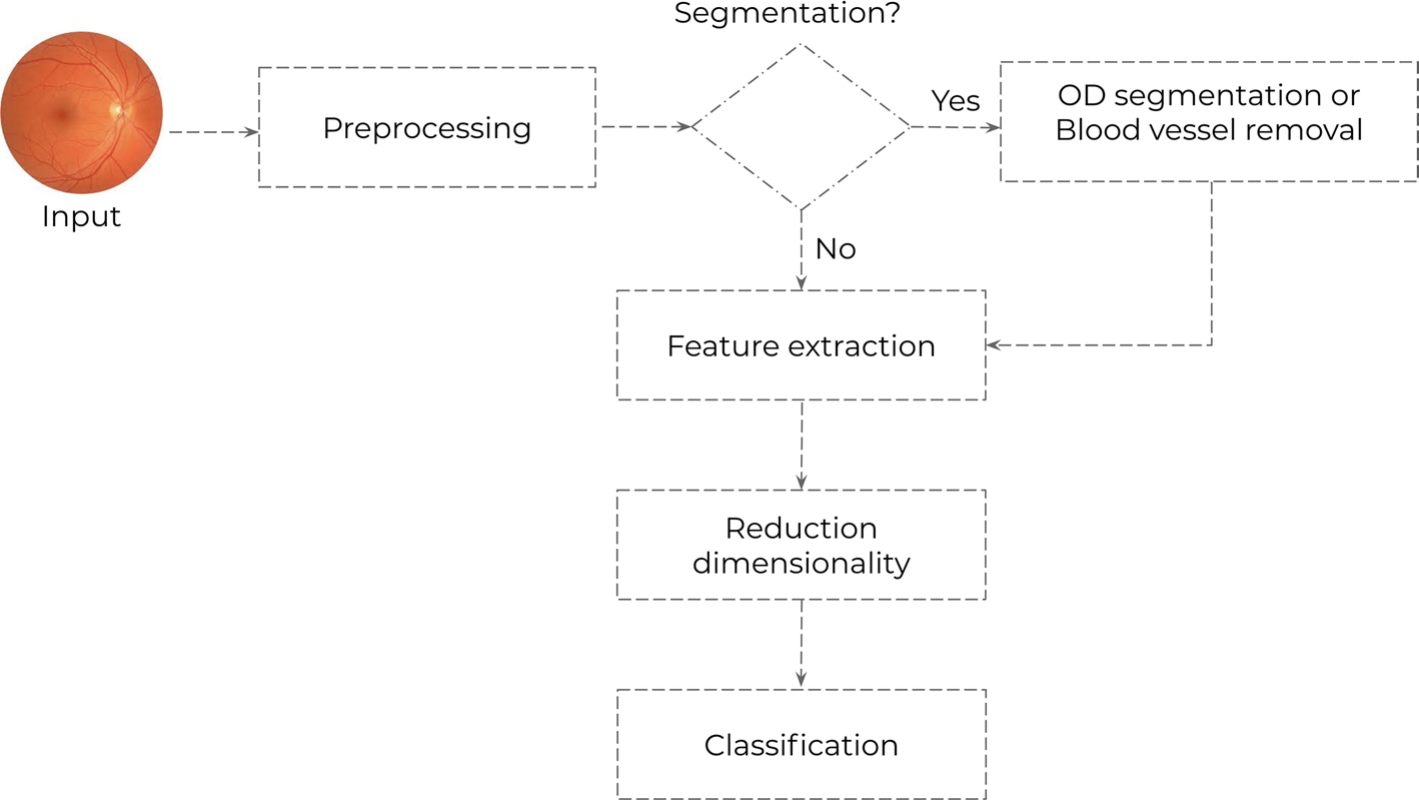
Generic architecture using features extraction

**Fig. 3:**
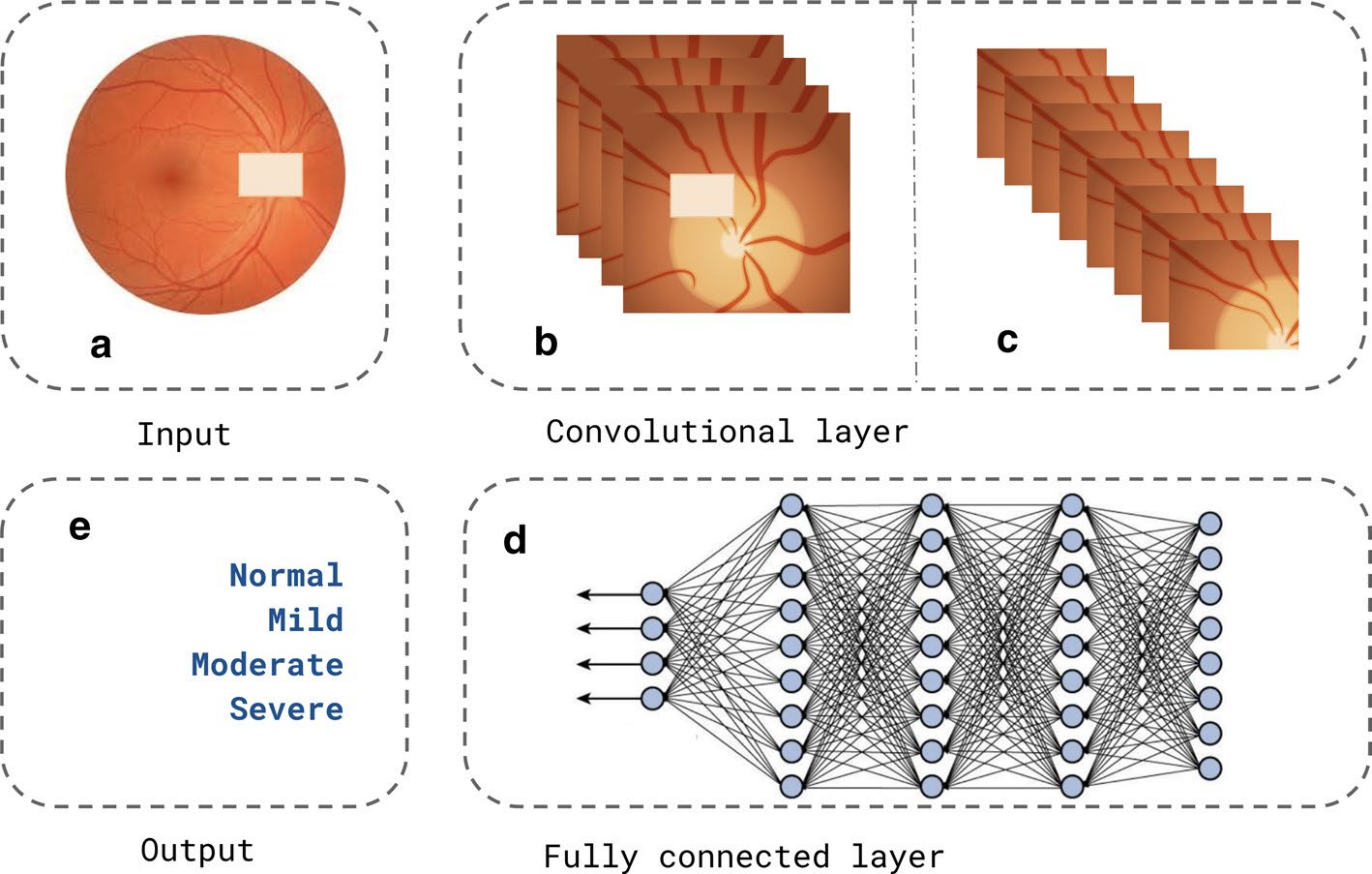
Generic architecture using deep convolutional network

Based on the selected studies, it was possible to discern two generic architectures for glaucoma diagnosis: generic architecture using feature extraction (represented in Fig. 2), and generic architecture using deep convolutional network (represented in Fig. 3).

In the researches that employed the feature extraction generic architecture, it was possible to describe a pipeline with five steps for the development method, such as preprocessing, feature extraction, dimensionality reduction and classification. The segmentation step emerges as optional. Yet the preprocessing is performed in different approaches: image size reduction [38–40], image channels manipulation [30, 45, 50], histogram equalization and noise filtering [48], histogram of visual words [49] or bilateral filtering [51]. All the techniques were applied in order to highlight the OD and the OD-cup region. Furthermore, an important feature in the retinal images is characterized by the brightness in the central region, as well as the high noise level in the edge region [74]. Still, all the techniques aim to improve the image’s quality through filtering and lighting enhancement. Additionally, such a process removes the image’s noise in order to reduce the processing time during the image analysis.

In the segmentation process, Issac et al. [40] segmented the OD and OD-cup using the red channel. In this manner, the morphological dilatation was the differential of this process. Such a method was performed to remove the possible gap in the segmented OD limit. Thus, it employed an element of dimension equal to the width of the primary blood vessels lying on the ONH, leading to a better OD segmentation.

However, Rehman et al. [51] performed the mentioned process utilizing the superpixel segmentation. Its main objective was to find similar pixel group clustering and to label those as the same type. It was observed that the processing step was facilitated due to the segmentation, which splits the images in regions. Differently, Singh et al. [47] segmented and preprocessed the OD in only one step. In the proposed method, the authors considered clinical assumption that the OD is the brightest region in the image [6]. Their algorithm localizes the OD employing the red channel to build a plane with various (*x*, *y*) points, since it allows OD localization and extraction.

Feature extraction is one of the most important steps in fundus imaging processing. Acharya et al. [39] extracted statistical metrics as the image features. Thus, the extraction of 32 features, considered importantly related to the image intensity, was performed. In retinal imaging, those are useful during the classification process. Moreover, a valuable contribution of Acharya et al. [39] was represented by the development of a risk index for glaucoma screening, which can be used in clinical daily activities. In addition, Raja et al. [45] employed almost the same metrics as Acharya et al. [39] with exception to skewness and kurtosis, extracted from the HWT.

The feature extraction is a key procedure when it comes to allowing the usage of classification algorithms and the reduction of data dimensionality. The method developed by Soltani et al. [48] is different, since it vertically and horizontally divides the OD and OD-cup diameters to compute the ISNT measure values and eye asymmetry. Such an aspect leads to the extraction of four characteristics, which are well known by ophthalmologists.

Differently, Mohamed et al. [50] provide information such as thickness, smoothness and regularity. These metrics indicate the relation among the pixel intensity values. That said, the SPL method was adopted to analyze the spatial distribution of gray values. Thus, this process occurs from the extraction of significant features within superpixels of the best color channels in order to derive a set of statistics for those features. Regarding this technique, the use of color channels represents a relevant aspect to be taken into consideration. Table 2 summarizes all applied methods for feature extraction, as well the number of used features throughout each classification method.

Some methods investigated for this review have applied a dimensionality reduction process [38, 47]. In this way, its purpose is to reduce the number of features in order that the classifier mediation and the precision costs may be decreased. The better and more important the features are, the faster the classifier will be, avoiding the dimensionality curse.

Singh et al. [47] used the PCA, while Noronha et al. [38] the LDA. The difference between these methods is that the LDA classifies the data, while the PCA changes the localization shape of the original database, projecting those in other space. Another feature of LDA is that it maintains the class separation [38]. Additionally, the utilization of the feature ranking using Fisher’s discrimination index (*F*) represents a considerable element regarding Noronha’s method. Contrary to further methods, Maheshwari et al. [30] use a feature normalization process. Hence, these researchers used the z-score to improve the classifier performance.

The classification process marks the ultimate step, described in Fig. 2. Furthermore, the evaluated papers made use of the supervised learning standard based on the training process from a labeled dataset that acquires a required function. Still, the data employed in the training were extracted from the images. The SVM classifier presented the most favorable results in ten of the papers selected, as in the research of the following authors: Acharya et al. [39], Issac et al. [40], Raja et al. [45], Mohamed et al. [50], and Rehman et al. [51].

The principal features of the support vector machine (SVM) method are its generalization and robustness in high dimensions. Apart from presenting a well-established theoretical basis into mathematics and statistics, that makes its usage easier, SVM can be employed for image classification. This method applies the high-dimensional linear hypothesis to produce hyperplane by measuring its margin, and searches for maximum points as well [75]. As for the classification problem performed by Soltani et al. [48], the authors use the fuzzy logic approach. Moreover, the SVM method needs an interval of values to indicate if a determined possibility is true or false.

To ascertain whether a diagnosis is correct, many parameters are needed. Accordingly, for developing a system design, the subsequent aspects must be considered: three linguistic variables, two inputs and one output; membership functions, which indicates normal, glaucomatous and glaucoma-suspect classes; and fuzzy rules, including a total of six. Beyond that, such rules are based on the idea that it is necessary to evaluate its previous versions, considering it to those following. In this way, the decision process of the classes’ labels is performed by using a decision-making logic which is based on clinical and imaging data.

The generic architecture that employs a deep convolutional network, as expressed in Fig. 3, consists of several layers of processing, which are trained to represent data in several levels of abstraction. In case the responsible model for this task consists of a cascade of processing layers that resemble biological processes, it is unnecessary to extract the image features [76]. Thus, such a model transforms the raw input into output through a function. That said, the architecture is basically constituted of an input (Fig. 3a), a convolutional layer (Fig. 3b, c), which is responsible for the feature extraction process, a fully connected layer (Fig. 3d), responsible for classification; and a desirable output (Fig. 3e). Differently from the architecture described in Fig. 2, the feature extraction and classification processes are performed by the model.

**Fig. 4:**
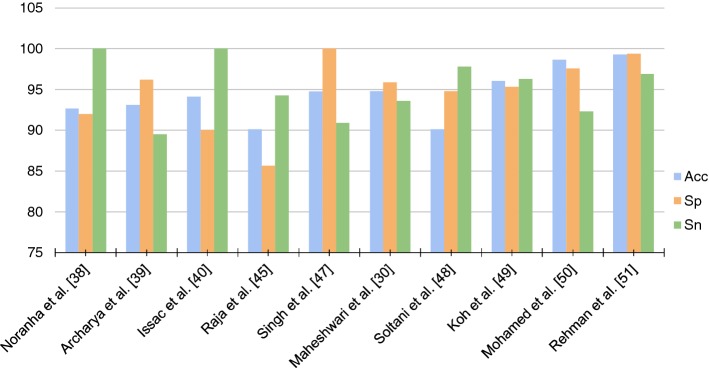
Metrics generic architecture using features extraction

In the method of Raghavendra et al. [62], four batch normalization layers were applied to allow the flow of the normalized data into the intermediary layers. Such a fact favors higher learning rates, which causes the process to be faster. In addition, a soft-max layer that allows reducing outliers in the data sample was inserted after the fully connected layer. Hence, Li et al. [55] trained a net with 22 layers. The highlight of this model is characterized by the use of mean and maximum clustering and concatenation, which leads to the increase of the net precision power.

However, some works showed variability regarding the generic structural application, as presented in Fig. 3. Dos Santos Ferreira et al. [63] developed an architecture based on U-net [59] solely to perform the OD segmentation. In this way, the net does not have a fully connected layer. The possibility of training all three color channels of the network is the key highlight of the mentioned work.

On the other hand, Christopher et al. [65] utilized a method that is being widely used in CNN on data argumentation. It aims to increase the number and the type of variations of the training data. This process may result in a better net performance, creating more generalized models, since some types of image quality transformations and variations are enabled. Additionally, it produces a considerable number of examples to be learned by the model, facilitating the correct image classification.

**Fig. 5:**
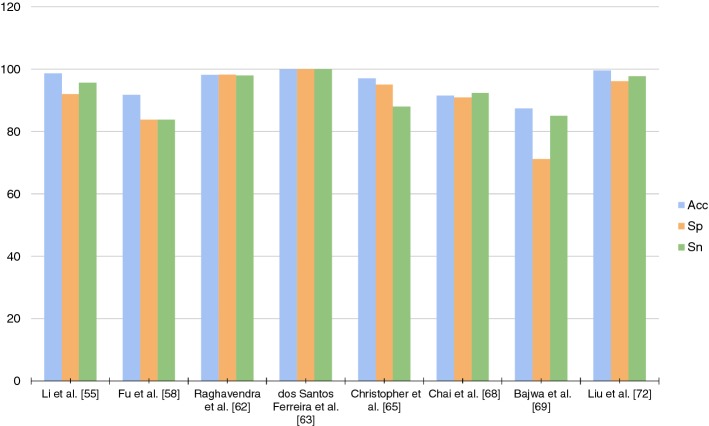
Metrics generic architecture using deep convolutional network

Other works, such as the study developed by Fu et al. [58], contain four deep streams corresponding to various levels and modules of the fundus image. This model takes into consideration various levels and modules of the fundus image, providing a segmentation-guided network which localizes a region of the disc and generates the disc segmentation representation.

The research of Bajwa et al. [69] proposed a framework composed of two levels: one for the OD localization and the other for classification. Moreover, the development of a new database represented another contribution of the authors [58]. Correspondingly, Liu et al. [72] developed a large-scale database of fundus images for glaucoma diagnosis (FIGD database). As for Chai et al. [68], their work took advantage of both deep learning models and domain knowledge. Aiming to evaluate all the images, the OD, and the domain feature, the authors projected a multi-branch neural network model. The differential of the method was that it had applied knowledge features—as CDR, RNFLD, PPA size, and symptoms from images and texts in the deep learning model—not only in the image, but also in domain.

Another substantial aspect observed throughout the analysis of the works is that all methods validated their models using the metrics of Sensitivity (Sn), Specificity (Sp), and Accuracy (Acc). In that manner, these metrics are key to recognition of the quality of created models. Figures 4 and 5 point out a graph with all these metrics. When training a model in the red channel, Dos Santos Ferreira et al. [63] obtained 100% in the three measures. However, in other channels as the blue channel, the Acc and Sn values were of 94% and 80%, respectively. Furthermore, the red channel presented better texture properties in the original images of the retina. In the images segmented by the method, this characteristic maintained such tone. Liu et al. [77] obtained the best Acc among the researched works: a Acc of 99.6% when applying deep learning. Thus, the development of a new database was of significant contribution. The method was evaluated on 274,413 images, representing the largest data set among the researched works.

With reference to the training and validation processes, some methods [38, 49, 65, 69] used cross-validation to train their model. Still, the most satisfactory results were achieved with its application. Noronha et al. [38], Koh et al. [49], Christopher et al. [65], Bajwa et al. [69] obtained the respective values for Acc: 92.65%, of 96.05%, 97%, and of 87.4%. Lastly, the most significant advantage of cross-validation is that instead of utilizing just one test set to validate the model, many other sets are created from the same data.

## Conclusion

The glaucoma severity is highly proportional to the optic disc cup’s enlargement, even when there is no direct association degree between these two features. The methods which used some machine learning techniques suggested that the CDR metric and the ISNT are substantial information for glaucoma diagnosis. Moreover, all the works indicated that the advantage of developing an automated method for ocular structure analyses is to decrease the variability within medical expertise agreements.

All the authors highlighted the significance of the development of CAD systems in order to diagnose the disease in the initial stages, since it increases the efficiency of the screening process. Each evaluated technique in this review diagnoses glaucoma in a generic way and does not take into account its variations. Hence, improving the diagnosis efficiency and developing computational methods to correctly classify the glaucoma variations are some challenges for future researches.

Based on the papers it was possible to distinguish two features: generic architecture using features extraction, and generic architecture using deep convolutional network. The architectures which applied the feature extraction, normally utilized an important step on its process, represented by dimensionality reduction. It is noteworthy to clarify that if such a step was excessive, the classifier could lose its generalization power. Thus, it is necessary to analyze the classifier behavior. Another aspect to be highlighted is that, among the works that employed such an architecture, there was not a predefined number of features to be used. This process depends ever on the developed method and on the tests performed throughout the validation process. Furthermore, the ideal dimensionality for a determined classifier and its dataset will be estimated by the model. Accordingly, these metrics are essential for the evaluation procedure of the models of the method.

According to Figs. 4 and 5, it is not possible to verify an increasing standard in the metrics values throughout the years. It appears that those values are more correlated with the chosen workflow and data than with the work’s originality, as well as the advance of new methods. In the previous section, the researchers were described in chronological manner. Still, those were weighted according to its originality and to the journal impact factor, since it can be considered as the work’s influence over new methods. Once the result values provided by the metrics did not allow the perception of a specific quality criterion, this aspect in question may be defined according to the database size, the variability, and to how the metrics were acquired.

The researches that employed a reduced dataset are more prone to obtain unfavorable results if applied on unpredicted data. This happens because small databases are more likely to accept smaller data variability. Thus, the dataset size portrays a meaningful role when it comes to the result confidence. Such a problem can be addressed by the evaluation of the performed methods in small databases.

Currently, deep learning is considered the state-of-the-art regarding computational vision and fundus imaging processing. Its differential is such a model is more flexible for the decision in how the data will be handled to generate the best result. In this way, such a net may acquire methods to maximize the ability of the net to distinguish, in a supervised case, the distinct classes in the images applied in the training process. Nonetheless, the disadvantage of deep learning is that an appropriate result is necessary in order to achieve an extensive database and high computational power for the processing.

There are several architectures for the CNN. Each of these is different in specific aspects such as: number and size of the layers, activation function, and net depth. In this way, it is not possible to determine the most efficient architecture for the glaucoma classification. Nonetheless, the empirical test demonstrated to be the best manner to perform the task. Although the differences between both generic architectures were evidenced, the development of researches indicates it is possible to develop an automated screening system for the glaucoma diagnosis.

## Data Availability

Not applicable
